# Engineering Resistance against *Sclerotinia sclerotiorum* Using a Truncated NLR (TNx) and a Defense-Priming Gene

**DOI:** 10.3390/plants11243483

**Published:** 2022-12-13

**Authors:** Patricia Messenberg Guimaraes, Andressa Cunha Quintana, Ana Paula Zotta Mota, Pedro Souza Berbert, Deziany da Silva Ferreira, Matheus Nascimento de Aguiar, Bruna Medeiros Pereira, Ana Claudia Guerra de Araújo, Ana Cristina Miranda Brasileiro

**Affiliations:** 1 Embrapa Genetic Resources and Biotechnology, Brasilia 70770-917, Brazil; 2 National Institute of Science and Technology (INCT Plant Stress Biotech), Brasilia 70770-917, Brazil; 3 INRAE, Institut Sophia Agrobiotech, CNRS, Université Côte d’Azur, 06903 Sophia Antipolis, France; 4 Department of Phytopathology, University of Brasilia (UnB), Brasilia 70910-900, Brazil

**Keywords:** wild *Arachis*, white mold, gene pyramid, expansins, immunity, TIR-NLR

## Abstract

The association of both cell-surface PRRs (Pattern Recognition Receptors) and intracellular receptor NLRs (Nucleotide-Binding Leucine-Rich Repeat) in engineered plants have the potential to activate strong defenses against a broad range of pathogens. Here, we describe the identification, characterization, and in planta functional analysis of a novel truncated NLR (TNx) gene from the wild species *Arachis stenosperma* (*AsTIR19*), with a protein structure lacking the C-terminal LRR (Leucine Rich Repeat) domain involved in pathogen perception. Overexpression of *AsTIR19* in tobacco plants led to a significant reduction in infection caused by *Sclerotinia sclerotiorum*, with a further reduction in pyramid lines containing an expansin-like B gene (*AdEXLB8*) potentially involved in defense priming. Transcription analysis of tobacco transgenic lines revealed induction of hormone defense pathways (SA; JA-ET) and PRs (Pathogenesis-Related proteins) production. The strong upregulation of the respiratory burst oxidase homolog D (RbohD) gene in the pyramid lines suggests its central role in mediating immune responses in plants co-expressing the two transgenes, with reactive oxygen species (ROS) production enhanced by *AdEXLB8* cues leading to stronger defense response. Here, we demonstrate that the association of potential priming elicitors and truncated NLRs can produce a synergistic effect on fungal resistance, constituting a promising strategy for improved, non-specific resistance to plant pathogens.

## 1. Introduction

In order to avoid diseases, plants have evolved an elaborate innate immunity that involves a two-layered defense system consisting of PTI (patterns-triggered immunity) and ETI (effector-triggered immunity) [1,2]. PTI requires pattern recognition receptors (PRRs) which are located on the host cell surface to recognize pathogen/microbe-associated molecular patterns (PAMPs or MAMPs) and trigger the basal immune responses. Host-adapted pathogens however, can interfere with PTI via small secretory proteins, known as effectors, and overcome this resistance. Hence, to recognize pathogen effectors, plants have evolved a repertoire of intracellular immune receptors, the NLRs (nucleotide-binding and leucine-rich repeat LRR domains) that mediate the specific recognition of pathogen effectors and initiate ETI [2]. Resistance associated with ETI can often lead to a plant hypersensitive response (HR), a mechanism typically accompanied by programmed cell death [3].

While the initial events of PTI responses mostly activated by PRRs have been intensively studied [4,5], detailed knowledge of ETI signaling initiated by NLRs has only recently been disclosed, with the determination of the “resistosome” structure [6,7], and the identification of “sensor” and “helper” NLRs [8,9] contributing to a better understanding of early ETI developments.

Additionally, until very recently, PTI was considered to contribute little to ETI. Nonetheless, the latest studies [10,11] show that although the two systems have different activation mechanisms and require different early signaling components, intricate interactions between PRR-mediated and NLR-mediated signaling cascades, and common signaling components exist, with PTI and ETI contributing in a collaborative manner to ensure effective immunity [12,13].

Canonical plant NLRs are composed of a central nucleotide-binding site (NBS) and a C-terminal LRR domain. The NBS domain functions as a molecular switch for NLR activation through nucleotide-dependent conformational changes, whilst the LRR domain is related to pathogen effector recognition [14]. Depending on the N-terminal domain, NLRs can be divided into two main classes: those having a toll and IL-1 receptor (TIR) domain and those with a coiled-coil (CC) domain, with a less frequent N-terminal RPW8 (resistance to powdery mildew 8) domain class also identified [15]. In addition to their conserved multidomain NLR architectures, NLRs lacking one or more of the canonical domains, commonly termed truncated NLRs, can also be functional and are found in various plant species [16,17]. NLRs can also contain unconventional domains, known as integrated domains (IDs), that can interact physically with their corresponding effectors and act as decoys of effector targets, enabling them to specifically detect pathogens [18].

While there are many examples of NLR genes conferring high levels of resistance against biotrophic pathogens of different classes [19,20], few studies demonstrate their positive regulation in plant resistance against necrotrophic pathogens, with some reports even showing NLR proteins implicated in host susceptibility to necrotrophic fungal pathogens [21,22]. To date, the best known NLR gene involved in resistance against necrotrophic fungi is the *Arabidopsis* TIR class *Leptosphaeria maculans* 3 gene (*RLM3*), which, in addition to the hemibiotrophic fungus *L. maculans* [23], provides resistance against three necrotrophic fungi; *Botrytis cinerea*, *Alternaria brassicicola*, and *A. brassicae*. More recently, the overexpression of an NLR from *Triticum aestivum* gene (*TaRCR1*) was found to increase resistance against the necrotrophic fungus *Rhizoctonia cerealis* [24], whilst chickpea NLR genes were found to be potentially involved in resistance against *Ascochyta rabiei* [25].

Among the necrotrophic fungi, *Sclerotinia sclerotiorum* is remarkable for its extremely broad host range and its aggressive host tissue colonization, rapidly triggering plant cell death and causing devastating yield losses on a wide variety of crop plants [26]. To date, plant genes conferring complete resistance against *S. sclerotiorum* have not been reported, with natural plant populations exhibiting a continuum of partial resistance [22]. Genetic engineering for *S. sclerotiorum* control has, to date, focused on using inducible defense-related antifungal proteins (chitinases, glucanases, and polygalacturonases) [27,28], transcription factors (TFs) that activate pathogenesis-related protein (PR) genes, and salicylic acid (SA) regulators such as NPR1 [29], all with variable success. With regard to targets in the pathogen, host overexpression of enzymes capable of degrading oxalic acid (OA), which is involved in the fungal pathogenesis, or the silencing of fungal genes involved in virulence, have also been shown to increase *S. sclerotiorum* resistance in transgenic plants [30,31].

Recent studies have revealed new roles for NLRs in addition to classical *R* gene function, including the conditioning of broad-spectrum resistance, regulatory roles in tolerance responses to abiotic stresses, or roles as “helpers” for other NLRs [17,27,32]. Additionally, the application of truncated NLRs in enhancing immunity against different pathogens has been explored in various species [23,33,34]. Exactly how truncated TNLs function in the immune system remains unclear, but there are indications that they might form heterocomplexes with full-length TNLs or other proteins to mediate immune responses [35,36,37]. Whilst the overexpression of NLRs often results in autoimmunity and significant fitness costs [38], different mechanisms controlling transcript levels of truncated, helper, and “inhibitor” NLRs make them potentially engineerable to increase plant defenses while limiting their fitness costs [38].

The recently discovered short biotrophic phase in the lifestyle of *S. sclerotiorum* [39,40], the secretion of virulence effectors by this necrotrophic fungus (SsPINE1) that suppresses host-basal defenses [41], and the role of phytohormones SA, JA/ET (jasmonic acid/ethylene) in pathogen infection [42], reinforce the potential functionality of R genes, including truncated NLRs, in *S. sclerotiorum* control. Such findings offer new alternatives for the genetic engineering of hosts for *S. sclerotiorum* management, including the use of a dominant or semidominant *R* gene-mediated strategy for early-phase disease control [43].

Wild species constitute important genetic repertoires of genes conferring tolerance against biotic and abiotic stresses, having evolved to adapt during the course of evolution [44], with NLR genes constituting an essential component of the resistance mechanisms in such species [45]. *Arachis* wild species show high levels of resistance against foliar fungi [46], the root-knot nematode *Meloidogyne arenaria* [47,48,49], ultraviolet radiation (UV) [50], and are more tolerant to drought [51,52]. In our previous studies [51,52], we isolated and characterized an *expansin-like B* gene from wild *A. duranensis* (*AdEXLB8*), with overexpression-activated phytohormone signaling pathways and the antioxidative system, leading transgenic plants to a defense primed state that enhanced plant defense responses against both biotic and abiotic stresses.

In this study we showed the potential of genetic manipulation of truncated NLR genes to achieve broader resistance against pathogens, including necrotrophic fungi. Here, the overexpression of a truncated NLR gene (TNx) from wild *A. stenosperma* (*AsTIR19*), both singly and in a genetic pyramid with the priming-related *AdEXLB8* gene, led to increased resistance against *S. sclerotiorum* in transgenic plants. This study reveals the potential of genetic manipulation of TNx genes to achieve broader resistance against pathogens, in the context of the development of more productive and sustainable crops.

## 2. Results

### 2.1. Truncated NLR (TNx) Genomic Distribution, Gene Structure and Protein Domains

**Figure 1:**
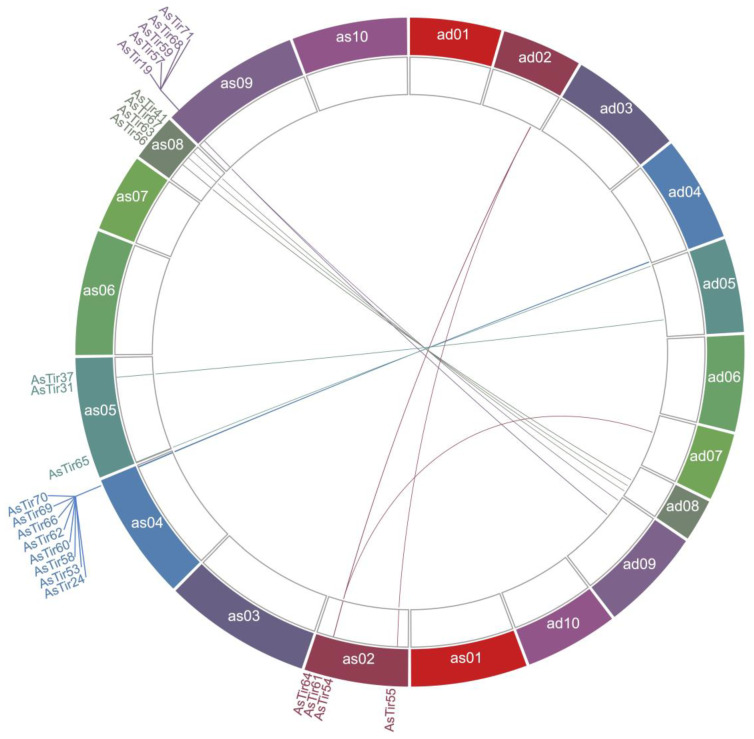
Genomic distribution of *A. stenosperma* TNx genes and their synteny with *A. duranensis*. Distribution of genes in ten chromosomes of *A. stenosperma* (as01–as10) and their orthologous in *A. duranensis* (ad01 to ad10). Colors vary with the chromossome numbers. The figure was generated by Circa software (https://omgenomics.com/ accessed on 10 August 2022). The TNx genes belonging the four clusters in *A. stenosperma* chromosomes as02, as04, as08 and as09 are highlighted.

Overall, 24 genes classified as truncated NLRs (TNx) were retrieved from the recently available representative genome of *A. stenosperma* (Supplementary Table S3). These genes are distributed in five out of ten chromosome pairs of *A. stenosperma* (Figure 1). The majority of the genes are located on chromosomes as04 and as09, in clusters of eight and five genes, respectively. The genomic distribution of the TNx genes shows high levels of synteny between *A. stenosperma* and *A duranensis* (Figure 1), and emphasizes the close association between AA genomes of *Arachis* [53]. One single exception is the *AsTIR54* gene located on chromosome as02 in *A. stenosperma*, present in chromosome ad07 in *A. duranensis* (Figure 1). In accordance with previous wild *Arachis* genome-wide studies [49,54], the majority of *A. stenosperma* TNx genes are located in clusters, and restricted to the distal chromosomal regions. Interestingly, three co-located genes on chromosome ad02 were found at both extremes of the chromosome as02, possibly due to a duplication event in *A. stenosperma* (Figure 1). Chromosome as04 harbors the biggest TNx gene cluster with eight genes, followed by chromosomes as09 and as08 containing five and four TNx genes each.

**Figure 2:**
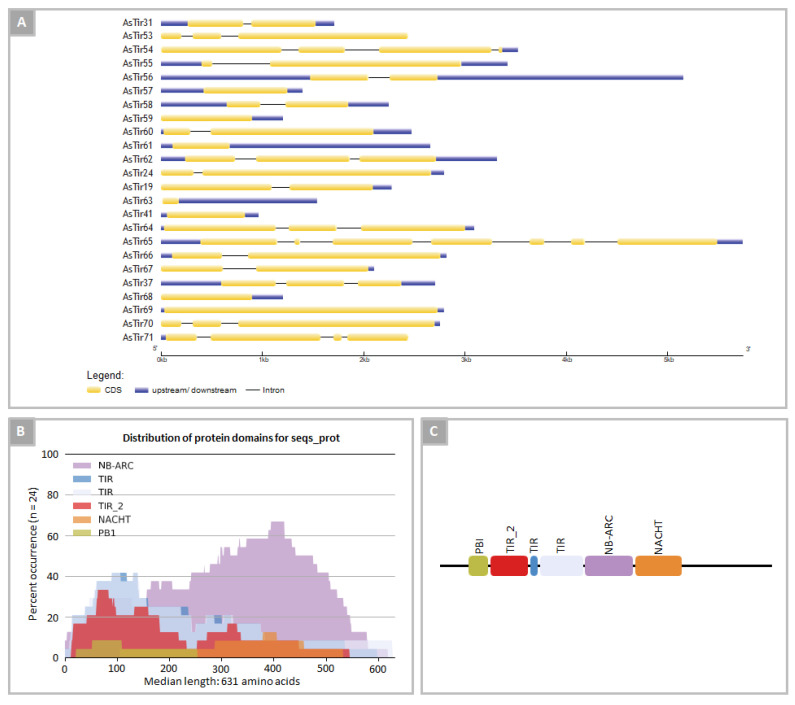
Gene and protein organization of TNx genes in *A. stenosperma*: (**A**) exon/intron organization of 24 TNx genes; (**B**) percentage of occurrence of conserved protein domains in TNx predicted proteins; (**C**) organization of conserved protein domains in TNx predicted proteins.

The exon-intron organization of the 24 *A. stenosperma* TNx genes varied, with genes showing from two to nine exons (Figure 2A). The protein structures showed conserved PFAM domains for truncated TNx [16] consisting of the highly conserved NB-ARC and at least one TIR domain (Figure 2B). Two of these proteins, AsTIR19 and AsTIR57, showed an additional potential integrated domain (ID) PB1 domain (Figure 2B,C), which is a protein interaction module that facilitates protein oligomerization and plays a role in many critical cell processes [55]. Another conserved domain found in *A. stenosperma* TNx proteins was NACHT (AsTIR41, AsTIR37, AsTIR31), a frequent NLR family-associated domain associated with the sensing of microbial products [56]. The ID PB1 is typically localized at the N-terminal region of the predicted *A. stenosperma* TNx proteins, while the NACHT domain is located at the C-terminal (Figure 2C).

### 2.2. TNx Genes in A. stenosperma Are Responsive to Biotic and Abiotic Stresses

**Figure 3:**
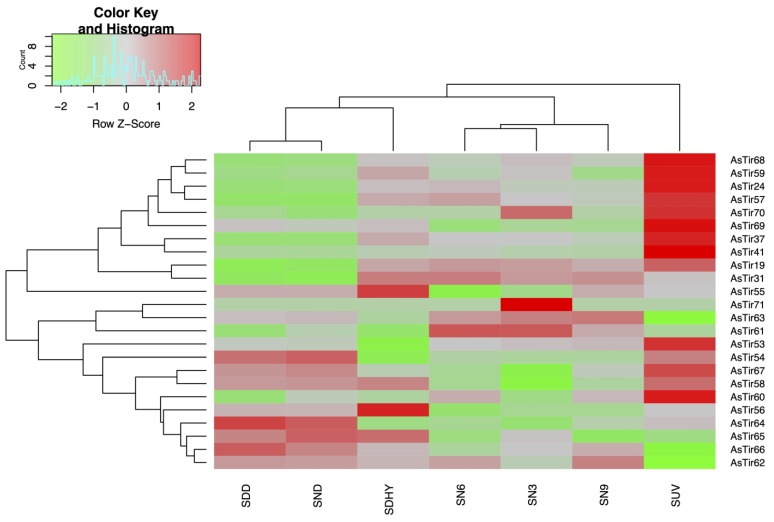
Heatmap of the relative RNA-Seq expression of 24 TNx genes from *A. stenosperma* plants subjected to biotic and abiotic stress treatments, with normalized log_2_FC values in a red-green scale (FDR < 0.05; log_2_FC). Plants submitted to: dry-down imposition (SDD); combined drought and nematode stresses (SND); dehydration treatment (SDHY), nematode infection for three, six and nine DAI (SN3, SN6, SN9) and UV exposure (SUV).

In order to evaluate the expression behavior of the 24 TNx genes in *A. stenosperma*, we analyzed the RNA-Seq data comprising transcripts of *A. stenosperma* plants submitted to dry-down (SDD), combined drought and nematode stress (SND), dehydration (SDHY), nematode infection at three, six and nine DAI (SN3, SN6, SN9), and UV exposure (SUV) (Supplementary Table S1) (Figure 3).

The majority (83%) of the 24 *A. stenosperma* TNx genes showed significant differential expression (DEG) when compared to control samples in at least one of the stresses analyzed, with only four exceptions (*AsTIR62*, *AsTIR65*, *AsTIR56*, *AsTIR64*) (Figure 3). UV exposure is the stress treatment that induced the greatest number of differentially expressed TNx (14), with an expression magnitude varying from −3.21-fold (*AsTIR63*) to 6.72-fold (*AsTIR41*), followed by 11 TNx significantly responsive to nematode infection (from—5.2-fold to 2.52-fold) in *AsTIR55* and *AsTIR71,* respectively (Figure 3). The TNx DEGs most upregulated during the dehydration treatment were *AsTIR55*, *AsTIR56* and *AsTIR65*, whilst three TNx were identified as DEGs under dry-down imposition, all showing downregulation (*AsTIR61*, *AsTIR19*, *AsTIR31*). Four of the five TNx genes located in chromosome as09 (*AsTIR68*, *AsTIR59*, *AsTIR57*, *AsTIR19*) showed similar regulation patterns under different biotic and abiotic stresses (Figure 3), suggesting a concerted response of these defense genes against harmful agents.

*AsTIR19* was the only TNx identified as a DEG under all the stresses tested (Figure 3), with relative expression ranging from −1.21-fold in roots submitted to dry-down imposition to 2.90-fold in leaves exposed to UV-C, which partially mimics the response to biotic stress. In addition, *AsTIR19* was previously reported as a significantly upregulated gene in response to inoculation with peanut late leaf spot fungi (*Cercosporidium personatum*) [46] and *M. arenaria* [49]. Given this, *AsTIR19* was selected for further functional validation against different biotic stresses in this study.

**Figure 4:**
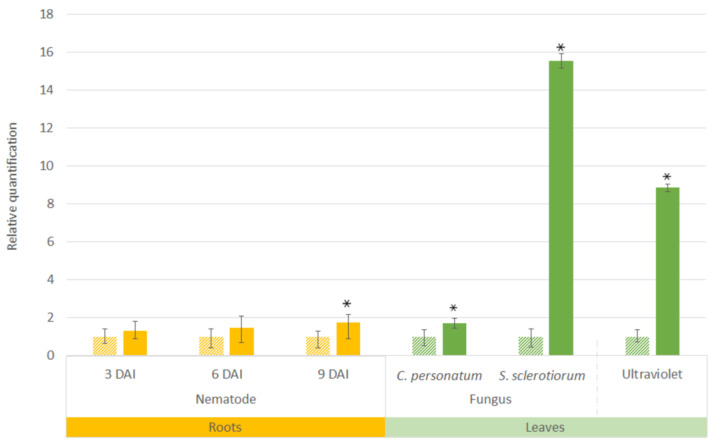
Relative expression of *AsTIR19* gene in *A. stenosperma* plants submitted to four biotic stresses. Hatched bars: roots and leaves from non-stressed control plants; Solid bars: roots inoculated with nematode (*Meloidogyne arenaria*) at three, six, and nine DAI and leaves infected with fungi (*C. personatum* and *S. sclerotiorum)* or exposed to ultraviolet (UV) light. The Relative Quantification (RQ) of *AsTIR19* mRNA levels in stressed samples was normalized with non-stressed control samples and RQ values are means and standard errors of 18 individuals. * *p* < 0.05 compared to control plants (*t*-test).

Here, the *in silico* expression of *AsTIR19* under different biotic stresses (Figure 3) was validated by qRT-PCR analysis, with the TNx positively regulated in roots of *A. stenosperma* infected with *M. arenaria* (3, 6, and 9 DAI) and in leaves submitted to UV radiation, *C personatum* and *S. sclerotiorum* (Figure 4). This broad response of *AsTIR19* to various stresses has previously been observed for other NLR genes [57], including abiotic stresses, such as salt and drought stress [58,59]. Given its broad responsiveness to different biotic stresses, and strong upregulation during *S. sclerotiorum* infection (15-fold), *AsTIR19* was selected for in planta functional validation in transgenic tobacco (*Nicotiana tabacum*) plants.

### 2.3. AsTIR19 Codes for an NLR-ID Fused Protein

**Figure 5:**
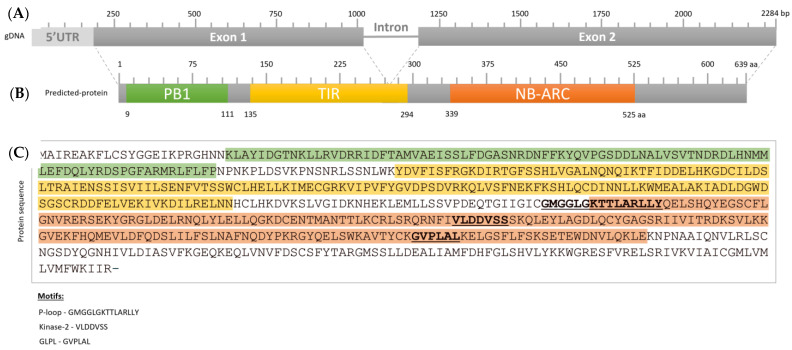
*AsTIR19* gene and predicted protein organization. (**A**) gene structure; (**B**) AsTIR19 protein domains; (**C**) AsTIR19 amino acid sequence.

We found that the *AsTIR19* gene structure consists of two exons of 824 and 1096 bp, and a single intron of 174 bp (Figure 5; Supplementary Figure S1A). The *AsTIR19* full coding sequence (CDS) of 1920 bp (Supplementary Figure S1B) was determined by aligning the *AsTIR19* genomic sequence with the best BLAST hits of *A. stenosperma* transcripts available at the NCBI (GenBank: EH043571; GenBank: GDBK01004898.1; GenBank: JR330906.1), followed by a detailed analysis using the software IGV [60].

*AsTIR19* showed high nucleotide conservation with the ortholog *A. duranensis* gene model Adura.G29LA (https://www.peanutbase.org/ accessed on 10 August 2022), coding for a putative NLR gene, with only 16 SNPs (single nucleotide polymorphism) between the two species. These SNPs lead to 5 synonymous and 12 non-synonymous amino acid substitutions, with three non-conservative substitutions (A/S, E/K and I/V) and nine conservative substitutions (Supplementary Table S4).

*AsTIR19* encodes a putative protein of 639 amino acids, with a theoretical pI of 6.92 and a molecular weight of 72.81 kDa, containing three domains situated at the following amino acid residue intervals: PB1_UP2 (9–111), TIR (135–294), and NB-ARC (339–525) (Figure 5; Supplementary Figure S1C). Two of these domains are typical of NLR resistance proteins. The first of these is a toll interleukin 1 receptor domain (TIR; Pfam: PF01582), which in plants is found almost exclusively in NLR resistance proteins as an intracellular signaling domain that mediates protein-protein interactions, playing a signaling role during resistance responses [61]. The second is a nucleotide-binding domain (NB-ARC; Pfam: PF00931), which is a functional ATPase domain present in all NLRs, involved in immunity and apoptosis [62]. The third domain in the AsTIR19 protein is a Phox/Bem1p (PB1) domain of type-2 (PB1; Pfam: PF00564), containing 89 amino acids in length and located at the N terminal (Figure 5). 

In addition to the typical TIR and NB-ARC domains, the AsTIR19 protein lacks the C-terminal LRR domain, and is therefore classified as a member of the TNx or TIR-X NLR protein family [63] (Figure 5). Although the majority of functional NLRs have both the NB-ARC and LRR domains, there are several reports of functional disease resistance genes encoding proteins that lack LRRs [57]. Likewise, recent studies demonstrated that NLRs with non-canonical domain architectures can carry Integrated Domains (IDs) that can perform sensor roles and enable the perception of pathogen effectors [18,64].

We also observed that the predicted PB1_UP2 and TIR-NBS fusion in the AsTIR19 protein structure was further supported by RNA-Seq read alignment data in wild and cultivated *Arachis* species (https://www.peanutbase/ accessed on 10 August 2022) (Supplementary Figure S6). The high transcript density spanning the corresponding fusion genomic region in *A. stenosperma* confirms that the detected fusion is real and not due to miss-assembly or annotation errors [64].

### 2.4. Overexpression of AsTIR19 and AdEXLB:AsTIR19 Reduces S. sclerotiorum Infection in Tobacco OE Lines

The *AsTIR19* gene functionality against the necrotrophic fungus *S. sclerotiorum* was evaluated here using tobacco overexpressing (OE) lines harboring *AsTIR19* singly or in a pyramid with *AdEXLB8*, a defense priming-related gene previously isolated by our group from wild *A. duranensis* [52]. Among the homozygous transgenic plants at T2 generation, two singly (TIR19-OE-1 and -2) and two pyramid (EXLB:TIR19-OE-1 and -2) OE lines were selected for *S. sclerotiorum* detached leaf bioassays. The overexpression of transgenes in the independent tobacco OE lines was determined by qRT-PCR for both transgenes (*AsTIR19* and *AdEXLB8*), with different expression levels observed (Supplementary Figure S3).

**Figure 6:**
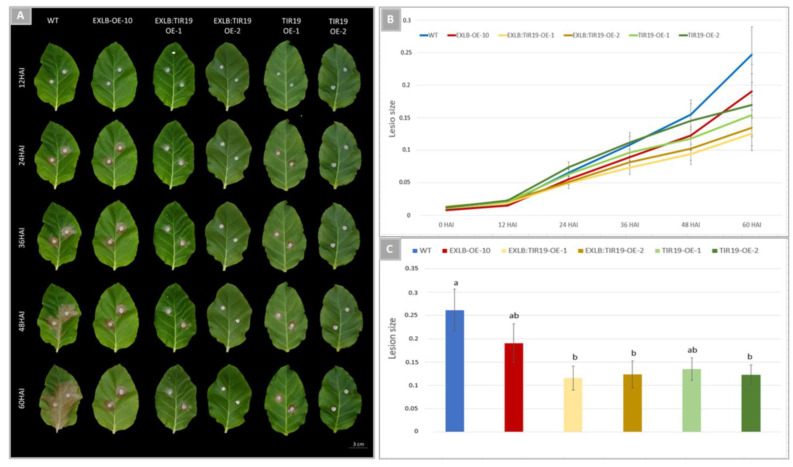
*Sclerotinia sclerotiorum* infection of detached tobacco leaves. (**A**) Lesion areas in leaves of WT, EXLB-OE-10, EXLB:TIR19-OE-1, EXLB:TIR19-OE-2, TIR19-OE-1, TIR19-OE-2 plants at 12, 24, 36, 48 and 60 h after inoculation (HAI); (**B**) Disease progress over 60 h, estimated based on the average ratio between the area of necrotic fungal lesions and total leaf area; (**C**) Average lesion area at 60 HAI in leaves of OE lines and WT plants. Values are means and standard errors of ten individuals, with different letters indicating significant differences based in ANOVA followed by Tukey’s test (*p* ≤ 0.05).

The lesion areas in fungal inoculated leaves were recorded at every 12 h after inoculation (HAI) up to 60 HAI. Differences in the disease development among genotypes were observed from 24 HAI onwards, with pyramid genotypes (EXLB:TIR19-OE-1 and -2) showing the smallest lesions across all analyzed time points (Figure 6A,B). Disease spots in WT and EXLB8-OE-10 leaves were well-developed by 48–60 HAI, whilst for the TIR19 and EXLB:TIR19 OE lines only reduced lesions were observed, which did not spread as extensively (Figure 6A,B). At 60 HAI, lesion areas on transgenic leaves were significantly smaller in both pyramids EXLB:TIR19-OE-1 (57%), EXLB:TIR19-OE-2 (53%) and in TIR19-OE-2 (50%) in comparison to WT leaves using ANOVA followed by Tukey’s test (*p* ≤ 0.05) (Figure 6C).

These results show that *AsTIR19* overexpression has a significant effect on the reduction in *S. sclerotiorum* lesions, and this effect can be enhanced by 13% when this NLR transgene is stacked with *AdEXLB8* (Figure 6C). This significant reduction in *S. sclerotiorum* lesion sizes in the pyramid lines in comparison with WT plants observed here was expected, as gene pyramids have been investigated experimentally for a diversity of pathogens, with many reducing disease levels below that of the single best gene [65]. We suggest that the cell-wall expansin gene (*AdEXLB8*), previously identified as a gene involved in priming state induction [52], together with the TNx gene *AsTIR19*, induced different but complementary resistance mechanisms in the plant host, leading to a significant reduction in the infection caused by the necrotrophic fungus. Both genes were isolated from wild *Arachis* species and could be implicated in its natural resilience against pests and environmental hazards [48].

### 2.5. S. sclerotiorum Hyphae Growth in OE Lines

In *S. sclerotiorum* inoculated leaves of all OE lines (singly and pyramid), cotton blue stained areas surrounding the lesions were generally much smaller, or even non-detectable, than observed in WT plants, due to the low density of developing hyphae (Supplementary Figure S4). Alterations in the hyphal growth of *S. sclerotiorum* was observed across all OE lines collected at 10 and 14 HAI in comparison to WT (Supplementary Figure S4). Morphological irregularities such as short length and irregular branching of the hyphae, contorted offshoots in the increasing tops, and bifurcated tips were also evident in the OE lines. Additionally, appressoria with irregular cushions originating from misshapen hyphae, as well as an absence of multihyphal structure or appressoria were also apparent (Supplementary Figure S4). These observations reinforce that the overexpression of *AsTIR19* singly, or in a pyramid with *AdEXLB8*, cause interferences in *S. sclerotiorum* hyphal growth during the first hours of the plant-pathogen interaction.

### 2.6. qRT-PCR Analysis of Marker Genes for Hormonal Defense Pathways in Tobacco OE Lines

**Figure 7:**
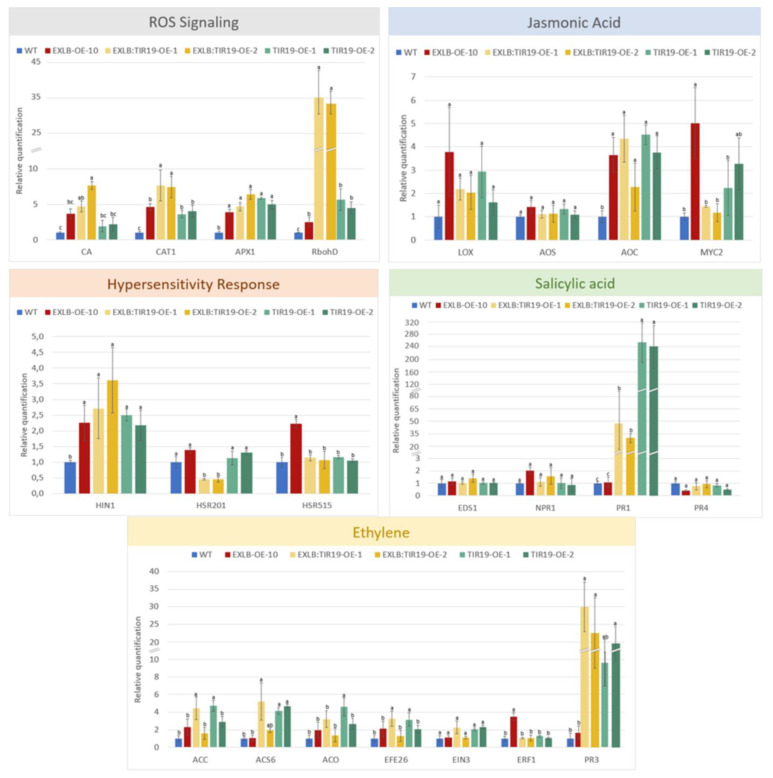
qRT-PCR expression analysis of OE lines. Relative quantification of mRNA levels of 22 tobacco marker genes involved in plant hormonal (SA, JA, ET) and defense pathways (ROS and HR) in singly and pyramid OE lines relative to (wild-type) WT plants. WT (blue); EXLB8-OE-10 (red), EXLB:TIR19-OE-1 (light yellow), EXLB:TIR19-OE-2 (dark yellow), TIR19-OE-1 (light green) and TIR19-OE-2 lines (dark green). Relative Quantification (RQ) values are means and standard errors of 18 individuals, with different letters indicating significant differences based in ANOVA followed by Tukey’s test (*p* ≤ 0.05).

Recent studies have shown that the interplay between SA and JA-ET and other molecules enables plants to defend themselves against both biotrophic and necrotrophic pathogen lifestyles [66,67]. Here, we analyzed the expression of 22 tobacco marker genes involved in biosynthesis, signaling, and interactions, particularly in SA, JA and ET pathways (Supplementary Table S2) in the transgenic OE lines and WT plants. These results showed that the single overexpression of *AsTIR19* in the tobacco OE lines led to an induction of the SA pathway, with the hallmark gene *PR1* (Pathogen Related Protein 1) showing strong upregulation (over to 200-fold) in relation to control WT (Figure 7-green bar). In consonance with SA induction, the HR marker gene *HIN1* was upregulated (over to 2-fold), (Figure 7: green bar) in the singly TIR19 OE lines. Concomitantly, the activation of the ET pathway, demonstrated by the strong upregulation of *PR3* (20-fold) and other ET biosynthesis precursors, together with the induction of components of the JA pathway in relation to WT control was also observed (Figure 7: green bar). In addition, reactive oxygen species (ROS) markers genes such as *CA*, *CAT*, *APX1*, and the NADPH oxidase *RbohD* were upregulated in TIR19 OE lines in relation to control WT (Figure 7: green bar).

Likewise, in the pyramid EXLB:TIR19 OE lines, ROS signaling was also clearly activated, as shown by the induction of genes involved in ROS homeostasis and antioxidant activities (*CA*, *CAT*, *APX1*) and the strong upregulation of *RbohD* (35-fold) (Figure 7: yellow bar), responsible for keeping the cell balance during ROS bursts [68]. Interestingly, the respiratory burst oxidase homolog *RbohD*, which elicits ROS production, was 7-fold more upregulated in the pyramid OE lines than in the monogenetic counterparts (Figure 7: yellow bar). RbohD is an NADPH oxidase enzyme that generates apoplastic superoxide ions (O_2_^−^), which have a crucial role in the outcomes of plant interactions with a wide range of pathogens and symbionts [69]. In the pyramid lines, the activation of ROS-mediated signaling pathway was accompanied by the upregulation of the HR marker genes *HIN1* and *HSR515*, whereas *HSR201* was clearly downregulated (Figure 7: yellow bar). As observed in TIR-OE lines, a significant upregulation of the SA and ET pathways, denoted by *PR1* (40-fold) and *PR3* (30-fold) markers, respectively, occurred in pyramid lines in comparison with the WT (Figure 7: yellow bar). Moreover, the activation of the ET and JA pathways in the pyramid OE lines was supported by the induction of other gene markers in each, respectively (Figure 7: yellow bar).

The expression behavior of marker genes in the EXLB-10 OE line reinforced our previous works [52], showing an increased expression of genes related to JA hormonal pathway, ROS detoxification and *RbohD* regulation (Figure 7: red bar). In addition, we demonstrated here that *AdEXLB8* overexpression activated the expression of some genes involved in HR (*HIN1*, *HSR201* and *HSR 515*) as well as in ET biosynthesis and signaling (Figure 7: red bar).

The phenotypic data and the expression of marker genes from different defense pathways here analyzed, suggest that both ETI and PTI immune responses might occur in the pyramid OE lines. In this case, the overexpression of the truncated NLR *AsTIR19* gene that induces the SA pathway and increases *PR1* production (250-fold), would possibly lead to ETI, whilst the concomitant overexpression of the defense priming-related gene *AdEXLB8*, which promotes ROS burst and the induction of JA-ET biosynthesis would promote PTI responses. The significant upregulation of the *RbohD* gene, which encodes a ROS-inducer NADPH oxidase, in the pyramid lines (35-fold) in comparison to the TIR singly lines (5-fold), suggests a converging role for this enzyme in pyramid plants (Figure 7). A hypothetical diagram showing the potential effects of the overexpression of *AsTIR19* and *AdEXLB8* in the pyramid lines leading to both ETI and PTI immune responses and their “cross-talk”, is described in Supplementary Figure S5.

Overall, the modified transcriptional dynamics of 22 marker genes from different defense hormone pathways observed here (Figure 7), reveals their involvement in the enhancement of the resistance observed in pyramid lines against the necrotrophic fungal pathogen *S. sclerotiorum*, without apparent negatively impacting the plant phenotype.

## 3. Discussion

Transcriptional differences and expression levels in well-defined comparisons have led to the identification of novel genes of defined function in many plant species [6]. In this study, the characterization and transcriptional analysis of truncated NLRs (TNx) in *A. stenosperma* under different biotic stresses led to the identification and isolation of a novel candidate gene against the necrotrophic fungus *S. sclerotiorum*. The overexpression of this TNx (*AsTIR19*) singly and in a pyramid with *AdEXLB*, another wild *Arachis* gene potentially involved in defense priming induction [52], increased the resistance against *S. sclerotiorum* by up to 57% in transgenic tobacco plants. To our knowledge, this is the first report on a truncated NLR engineered in transgenic plants conferring resistance to *S. sclerotiorum*.

### 3.1. AsTIR19 Is a Broad-Spectrum Responsive TNx

*AsTIR19* showed low basal expression in different *A. stenosperma* tissues (leaves and roots), with rapid upregulation in response to various biotic and abiotic stresses. Such expression may be expected, given that as NLR genes can potentially trigger cell death, they are often expressed at low levels in various plants unless elicited by pathogens [38]. More recently, it was demonstrated that NLRs can also be induced by environmental stresses, such as waterlogging, drought, heat, and cold, and therefore can be associated to general resistance responses to stressful conditions [59,70]. Hence, NLR expression requires a high degree of regulation at both the transcriptional level, involving methylation or involvement of miRNAs and siRNAs [71], as well as post-transcriptionally, at the protein level, where the functionality of receptor NLR proteins is determined by their interactions with other proteins, including other NLR proteins [72].

Within its native genomic background (*A. stenosperma*), *AsTIR19* expression has been shown to be significantly increased upon infection by different pathogens (*Meloidogyne* spp., *C. personatum* and *S. sclerotiorum*) and UV radiation, and also in response to abiotic stresses (drought, dehydration). This NLR broad-spectrum response has previously also been reported in *Arabidopsis*, where functional and non-functional NLR alleles respond to both types of stresses and are maintained in the genome due to a trade-off between biotic and abiotic stress adaptation, with polymorphisms in these genes being influenced by competing environmental stresses [73]. In addition to responding to different types of stress, some truncated NLRs can also trigger defense responses to organisms with different lifestyles, such as the well-known *RLM3* from *Arabidopsis*, which codes for a TNx protein involved in defense against both hemibiotrophic and necrotrophic fungi [23].

This widespread response of certain NLR proteins might be due to their indirect activation by the interaction of effectors with host proteins that they target, with such an indirect mode of recognition enabling a single NLR protein to mediate resistance to multiple types of pathogens [74], or to the sensor/helper model of NLR function [75,76]. In this later configuration, sensor NLRs are dedicated to detecting pathogen effectors, whilst helper NLRs are required to initiate immune signaling, potentially resulting in a hypersensitive cell death response (HR) [72].

Although here we demonstrated the role of *AsTIR19* in increasing resistance against *S. sclerotiorum*, the mechanisms that underline its function in resistance, such as being a potential regulator of resistance responses [77], or acting in concert with other sensor NLRs displaying different sensitivities to biotic and abiotic elicitors [72], has not yet been elucidated.

We also showed that the protein structure of *AsTIR19* shows, in addition to a TIR and NB-ARC conserved domains, contains a PB1_UP2 domain located at the N-terminal. Various proteins that harbor a PB1 domain undergo oligomerization [64], with two well-studied plant PB1 domain-containing proteins, AtNBR1 from *Arabidopsis* [78] and Joka2 from tobacco [79], known to be involved in autophagy in responses to stress. Selective autophagy plays an important role in broad plant responses to both biotic and abiotic stresses, with autophagy-deficient mutants being compromised in resistance to necrotrophic pathogens [80]. We suggest that the PB1_UP2 domain in the N-terminal of the AsTIR19 protein is an integrated domain (NLR-ID), potentially involved with oligomerization and pathogen recognition in *A. stenosperma*. Our RNA-Seq data supports this predicted fusion, as the transcript density of the PB1 domain in *A. stenosperma* roots under biotic and abiotic stresses is in concert with the other domains of *AsTIR19*. The protein-protein binding function of the PB1 domain in other stress-responsive proteins (NBR1 and Joka2), and the evidence that NLR and a host protein involved in indirect recognition can be fused together [18,76], reinforce this assumption.

The modifications of NLR-IDs as well as the replacement of IDs by other identified effector targets from the host genome have emerged as promising tools for the development of novel strategies of plant disease control [32]. Moreover, a recent study on *Magnaporthe oryzae* effectors and correspondent NLR receptors demonstrated the feasibility in the design of new effector recognition specificities in NLRs through molecular engineering of IDs [81].

### 3.2. AsTIR19 and AdEXLB8:AsTIR19 Induce Hormonal Defense Pathways in Tobacco OE Lines

Although there are many examples of NLR genes conferring high levels of resistance against biotrophic pathogens of different classes [19,20], few have been described as enabling resistance to necrotrophic pathogens, with *RLM3* from *Arabidopsis* [23], and *Rml2* and *LepR3* in *Brassica napus* that confers resistance to *L. maculans* [82,83], being the best well-known examples. In the present study, we showed that the single overexpression of *AsTIR19* led to an average reduction of almost 50% in lesion sizes of *S. sclerotiorum* in tobacco OE lines compared to WT plants, revealing that the activation of this unconventional NLR variant can indeed induce downstream signaling that leads to ETI and other defense responses. The further combination of *AsTIR19* and *AdEXLB8* overexpression in tobacco pyramid OE lines increased the reduction of *S. sclerotiorum* lesions up to 57%, showing an additive effect of transgene stacking in the pathogen resistance. 

Activation of the plant immune system can induce a series of defense responses, such as increased calcium concentration in the cytoplasm, ROS accumulation, callose deposition, activation of MAPKs, and pathogenesis-related (PR) genes expression [84]. Plant hormones (SA, JA and ET) and their crosstalk also play a crucial role in resistance against biotrophic and necrotrophic pathogens [85].

Here, a clear regulation of genes encoding PR proteins in the OE lines was observed, with *PR1* and *PR3* genes upregulated in both single and pyramid OE lines. *PR1* is a crucial marker gene in the SA pathway and systemic acquired resistance (SAR) and encodes a PR protein that displays antimicrobial activity against different pathogens [86]. In the pyramid lines, despite the lower expression levels of the *PR1* gene compared to single OE lines, a strong induction of the *PR3* gene was observed. *PR3* encodes a class I chitinase that hydrolyzes the fungal cell wall component chitin [87] increasing resistance to fungal infection [88]. We suggest that here, its upregulation can also be due to the alternate defense ET pathway [89], as other ET marker genes (*EF26*, *ACS*, *ACC*) were also upregulated in these plants.

In addition to *PR3* gene induction, the activation of the ET-JA signaling pathway also contributes to the enhanced level of resistance to the necrotrophic fungus *S. sclerotiorum* observed in the pyramid lines. Previous reports show that the overexpression of the transcription factor ERF, involved in ET biosynthesis, increased the resistance against necrotrophic fungi such as *Botrytis cinerea* and *Plectosphaerella cucumerina* in transgenic *Arabidopsis* [90]. Additionally, in rice, the overexpression of *ACS2*, an ET biosynthesis enzyme, increased the resistance against both necrotrophic (*Rhizoctonia solani*) and biotrophic (*M. oryzae*) fungi [91].

In consonance with *PR3* upregulation in response to ET-JA signaling, a ROS burst was observed in the pyramid lines, as denoted by the strong upregulation of *RbohD*, and also by peroxidase gene induction, likely also contributing to enhanced defense response against the fungus, with these molecules acting as local and systemic secondary messengers to trigger additional immune responses [92]. ROS can be directly toxic to pathogens or lead to HR in transgenic pyramid plants, as supported by the upregulation of HR marker genes (*HIN1*, *HSR515*). Such a response can prevent further pathogen spread during the early biotrophic phase of *S. sclerotiorum* [39,40], leading to even higher levels of resistance.

In parallel, NLR proteins such as AsTIR19 can mediate ET signaling and increase ROS production, as observed by the upregulation of ET markers and *RbohD*, contributing to an enhanced defense response to *S. sclerotiorum* in the single OE lines. Consistent with this hypothesis, the overexpression of an NLR from island cotton (*GbaNA1*) conferred resistance in *Arabidopsis* against the necrotrophic fungus *Verticillium dahliae*, accompanied by a significant enhancement of ROS accumulation and the expression of genes associated with the ET signaling pathway [93]. Likewise, in rice, the overexpression of the transcription factor OsBIHD1, which physically interacts with the NLR protein Pik-H4, was shown to regulate resistance against the hemibiotroph *M. oryzae* through direct activation of the ET signaling pathway [94]; and in wheat, the overexpression of the NLR protein TaRCR1, which regulates ROS-scavenging and production, significantly increased the resistance against the necrotrophic fungal pathogen *Rhizoctonia cerealis* [24].

Recently, Wei et al. [41] showed that *S. sclerotiorum* secretes an effector (SsPINE1) that directly interacts with and functionally inactivates polygalacturonase-inhibiting proteins, which are important basal defense proteins limiting fungal invasion. This virulence mechanism was proved to be conserved in a broad range of necrotrophic fungal pathogens, and reinforces the role of plant disease resistance proteins, such as NLRs, in counteracting necrotrophic pathogens in the early phases of the interaction.

### 3.3. RbohD Mediates ETI and PTI Defense Responses in Pyramid Lines

In this study, the increased production of ROS incited by *AdEXLB* overexpression in the pyramid OE lines, potentially leading to the acquisition of a defense stress primed state, most likely led to another layer of the defense response against *S. sclerotiorum*, given that these molecules also act as local and systemic secondary messengers to trigger additional immune responses [92]. We also observed a strong upregulation (5.5-fold) of the *RbohD* gene in the EXLB:TIR19 OE plants in comparison to their monogenetic counterparts.

This is in agreement with recent studies in *Arabidopsis* [10,11,12], which show that PRRs and NLRs can function synergistically to ensure a full active status of the membrane-localized RbohD, a key immune component, which in turn mediates ROS induced by ETI and full disease resistance. This PRR-mediated phosphorylation of the RbohD is a crucial early signaling event connecting PTI and ETI signaling cascades and is necessary for the full activation of RbohD during ETI in *Arabidopsis* [10].

Here, we also suggest that potential ETI-PTI cooperation in the pyramid plants occur (Supplementary Figure S5), in which *AdEXLB8* overexpression induces ROS, possibly contributing to an ETI-associated pathogen response analogous to that incited by PRRs involved with TNLs [11]. In this hypothetical model, *AdEXLB8* signaling would contribute to maximal phosphorylation of RbohD during ETI, while NLR signaling, activated by *AsTIR19*, would additionally increase the levels of RbohD (Supplementary Figure S5), highlighting the dual requirement of both *AdEXLB8* and *AsTIR19* signaling to ensure a more robust ROS production during ETI [10,11]. We also suggest that the strong *PR3* induction observed in the pyramid OE lines is a result of *AdEXLB8*-induced JA signaling, leading to the increased production of *PR3* via the ET-JA pathway in these plants (Supplementary Figure S5).

In summary, the strong upregulation of *RbohD* (35-fold) in the EXLB:TIR19 OE pyramid lines, associated with the expression behavior of genes involved with defense hormone pathways, ROS, and HR responses, suggests that *RbohD* is a crucial gene in regulating immune signaling, possibly acting as a hub that links PTI and ETI responses in these plants.

### 3.4. Novel Strategies for the Use of NLR Pyramids in Pathogen Control

NLRs have been widely used in biotechnological approaches to increase resistance against several pathogens, including a few necrotrophic and hemibiotrophic fungi. Nonetheless, some drawbacks associated with their use have been described, such as the faster evolution of pathogen avirulence genes in comparison to plant *R* genes, heterologous NLR genes being affected by the host plant immune system, or not being able to properly match the local immunity signaling pathway [95]. Therefore, new strategies for NLR employment are required, including the use of gene pyramids composed of genes with different modes of action and targets on the pathogen [65,96]. Our results suggest that the use of a combination of transgenes with complementary biological function, that potentially incites different immune responses (PTI and ETI), represents a promising strategy to broadly increase the effectiveness of NLRs against numerous diseases in crop plants, leading to more sustainable cultivars with improved resistance to necrotrophic pathogens. 

## 4. Materials and Methods

### 4.1. Genomic Distribution of TNx Genes in A. stenosperma

In order to identify truncated NLRs (TNx) genes in the *A. stenosperma* genome, we applied the NB-ARC domain (PF00931) for an HMMsearch against the *A. stenosperma* assembly from NCBI (PRJNA610652) and against the *A. duranensis* annotation of NLR genes from Bertioli et al. [97]. We compared *A. duranensis* sequences annotated as truncated NLRs (TNx) against the *A. stenosperma* genome, and only those with more than 80% of similarity were kept in this study. The gene structure of *A. stenosperma* TNx genes was represented using GSDS (http://gsds.gao-lab.org/ accessed on 10 August 2022), with the protein sequence submitted to DomainViz (https://uhrigprotools.biology.ualberta.ca/ accessed on 10 August 2022) for visualization of the distribution and organization of conserved PFAM domains. The location of the TNx genes on the ten *A. stenosperma* and *A. duranensis* chromosomes was represented using Circa software (https://omgenomics.com/circa/ accessed on 10 August 2022). 

### 4.2. Expression Profile of A. stenosperma TNx Genes in Response to Stress

The *in silico* expression behavior of the 24 predicted *A. stenosperma* TNx genes under different biotic and abiotic stresses was evaluated using previously produced transcriptome RNA-Seq data (Supplementary Table S1).

For the expression pattern analysis of the 24 TNx from *A. stenosperma* under five different stress treatments, we performed a heatmap visualization using the RNA-Seq data (Supplementary Table S1) as input for the gplots R package, as previously described [98]. The *A. stenosperma* TNx genes were considered as significantly Differentially Expressed Genes (DEGs) in each treatment when their relative gene expression levels had an adjusted *p*-value (false discovery rate; FDR) < 0.05 and at least 2.3-fold change (FC) value between stressed and control samples (log_2_FC > 1.2 or <−1.2).

### 4.3. qRT-PCR Expression Analysis of AsTIR19 in A. stenosperma Plants under Different Stresses

In order to evaluate expression via quantitative reverse transcription PCR (qRT-PCR) of the candidate gene *AsTIR19* in *A. stenosperma* roots and leaves submitted to distinct stressed conditions, we utilized the same biological material (roots and leaves) that were previously employed for RNA-Seq experiments (nematode infection, UV exposure) and 454/Roche (*Cercosporidium personatum* infection) (Supplementary Table S1). Material was maintained at −80 °C and RNA subsequently extracted using the same extraction protocol as employed for leaves infected with *S. sclerotiorum* agar plugs as described below. Total RNA from *A. stenosperma* was treated with DNAse, and reverse transcribed as previously described [99]. The expression behavior of *AsTIR19* in root and leaf tissues of *A. stenosperma* and under different stress treatments was further validated by qRT-PCR analysis, as described below. Both *60S* and *GAPDH* (Supplementary Table S2), were used as reference *Arachis* genes, in accordance with Morgante et al. [100].

### 4.4. AsTIR19 Characterization and Cloning

To determine the complete coding sequence of *AsTIR19*, we aligned the best BLAST hits from *A. stenosperma* available at the NCBI (EH043571; JR330906.1; GDBK01004898.1), and visualized the results using the default parameters of the online MultAlin tool (http://multalin.toulouse.inra.fr/multalin/ accessed on 20 August 2022). Polymorphisms amongst the above sequences were checked using the Integrative Genomics Viewer (IGV) (https://igv.org/ accessed on 20 August 2022). Isoelectric point and molecular weight of the AsTIR19-deduced protein were predicted using software available at ExPASy (http://expasy.org/ accessed on 20 August 2022).

The consensus *AsTIR19* coding sequence (1920 bp) was synthesized and cloned (Epoch Life Science Inc., Missouri City, TX, USA), under the control of the *Arabidopsis* actin 2 promoter (ACT-2) and the *Agrobacterium* nopaline synthase (NOS) terminator, at the *XhoI* restriction site of pPZP_BAR [101]. The resulting binary vector, pPZP-AsTIR19, also contained two additional cassettes for the constitutive expression of the enhanced green fluorescent protein (*eGFP*) reporter gene and the *bar* gene for glufosinate ammonium herbicide selection. The pPZP-AsTIR19 binary vector was then introduced into the disarmed *A.tumefaciens* strain ‘GV3101’ by a standard electroporation protocol. Transformed colonies were selected by PCR using primer pairs flanking the GFP, BAR or *AsTIR19* sequences (Supplementary Table S2). 

### 4.5. AsTIR19 and AdEXLB: AsTIR19 Overexpression in Tobacco Plants

Wild type (WT) tobacco plants cv. ‘Xanthi’ were transformed with *A. tumefaciens* harboring the binary vector pPZP-AsTIR19 using the leaf disc transformation procedure [102]. For pyramid transgenic plants, previously obtained hygromycin-resistant tobacco *AdEXLB8* overexpressing line OE-10 (at T3 generation) [52], were also transformed with the pPZP-AsTIR19 vector. Transgenic glufosinate-resistant seedlings at T1 generation were confirmed by PCR analysis using primer pairs flanking the *AsTIR19* and *AdEXLB8* sequences (Supplementary Table S2). Two independent single gene overexpressing (OE) transgenic lines (TIR19-OE-1 and -2) and two pyramid OE lines (EXLB:TIR19 OE-1 and -2) were selected for further bioassay analysis. The overexpression of *AsTIR19* and *AdEXLB8* transgenes was confirmed in the tobacco OE lines at T2 generation by qRT-PCR analysis, as described below using specific primers (Supplementary Table S2). 

### 4.6. S. sclerotiorum Bioassays in TIR19 and EXLB:TIR19 OE Lines

*S. sclerotiorum* detached leaf bioassays, fungal growth and inoculation, and symptom assessment were conducted essentially as described in Brasileiro et al. [52]. Overall, 20 detached leaves were evaluated for each OE-line. For each of the ten individuals of TIR19-OE-1 and -2; EXLB:TIR19-OE-1 and -2 OE lines, as well as for the controls EXLB-OE-10 and WT, two detached leaves of eight-week-old tobacco plants were placed on moisturized filter paper in large square dishes (500 cm^2^). Leaves were inoculated with fungal agar plugs (5 mm) according to Perchepied et al. [103] and maintained at 22 ºC in the dark for fungal lesion recording.

Images of tobacco leaves were recorded at 0, 12, 24, 36, 48 and 60 h after inoculation (HAI), and used to calculate the ratio between necrotic lesion area and total leaf area. The results represented the mean value of ten independent replicates and sample variability was indicated as the standard error of the means. One-way analysis of variance ANOVA followed by post-hoc Tukey’s test (*p* < 0.05) were employed to examine the differences between means.

### 4.7. Microscopy Analysis of S. sclerotiorum Infection in the OE Lines

Microscopy analysis was conducted in detached leaves of eight-week-old single gene and pyramid tobacco OE-lines, together with the controls EXLB-OE-10 and WT plants. Leaves were inoculated with *S. sclerotiorum* mycelial agar plugs as described above, and collected at 6, 10, 14, 18 HAI. After plug removal, a rectangular area around the lesions of each leaf was collected and immersed in EAF fixative solution (ethanol/acetic acid/formol/saline at 40:5:10:45 *v*/*v*) at 4° for 12 h. Samples were transferred to 70% (*v*/*v*) ethanol solution, maintained at 4 °C for an additional 12 h, then finally treated with lactophenol cotton blue for 12 to 24 h. Each leaf fragment was observed separately using both an M205 stereomicroscope (Leica Microsystems, Wetzlar, Germany) and an AxioPhot epifluorescence microscope (Zeiss, Oberkochen, Germany).

### 4.8. Hormonal and Transcriptional Regulation in OE Lines

Total RNA was extracted from a pool of six T2 seedlings of tobacco OE lines and WT plants using the TRIzol^®^ Reagent (Ambion^®^, Foster City, CA, USA), purified using the RNeasy Plant Mini Kit (Qiagen, Hilden, Germany), treated with DNAse, and reverse transcribed as previously described [99]. Basal expression of the transgenes (*AsTIR19* and *AdEXLB*) and the relative expression of 22 defense-related genes was determined in OE lines and WT plants through qRT-PCR analysis, as previously described [52]. qRT-PCR reactions were performed in three biological replicates on a StepOne Plus Real-Time PCR System (Applied Biosystems, Foster City, CA, USA) using specific primers (Supplementary Table S2). The online real-time PCR Miner tool [104] was used to estimate primer efficiency and optimal average cycle of quantification (Cq) values. The relative quantification (RQ) of target gene mRNA levels was normalized with two reference genes (*NtL25* and *NtActin*; Supplementary Table S2), and one-way analysis of variance (ANOVA) followed by Tukey’s test (*p* ≤ 0.05) were employed to examine the differences between means using the SATqPCR web tool (http://satqpcr.sophia.inra.fr/cgi/home.cgi/ accessed on 10 November 2022) [105].

## 5. Conclusions

The modular architecture of plant NLRs offers potential for genetic engineering towards durable and broad-spectrum resistance. Recent findings have revealed crosstalk and cooperation between ETI and PTI, with the association of both cell-surface (PRRs) and intracellular receptors (NLRs) in engineered plants having the potential to activate robust defense against a broad range of pathogens. Here, we demonstrated that the association of PTI elicitors and truncated NLRs, namely *AdEXLB8* and *AsTIR19*, produced a synergistic effect on *S. sclerotiorum* resistance, with *RbohD* being a crucial gene in regulating immune signaling, possibly acting as a hub that links PTI and ETI responses in these plants. As new resources and technologies such as resistance gene enrichment sequencing or targeted genome editing become available, our potential to discover new NLRs, including truncated NLRs, constitutes a promising strategy for genetic intervention that may lead to improved, non-specific resistance to plant pathogens.

## Supplementary Materials

The following supporting information can be downloaded at: https://www.mdpi.com/article/10.3390/plants11243483/s1, Figure S1: *AsTIR19*: (A) genomic sequence; (B) coding sequence (CDS); (C) protein sequence; Figure S2: sequence alignment of *A. duranensis* (Aradu. G29LA) and *A. stenosperma* (*AsTIR19*): (a) coding sequences (CDS) showing 16 SNPs; (b) predicted proteins showing 11 aminoacids substitutions; Figure S3: qRT-PCR analysis of basal expression of *AsTIR19* and *AdEXLB* transgenes in the five independent tobacco OE lines and wild-type; Figure S4: detached leaves of tobacco OE lines and WT stained by cotton blue and collected at 10 and 14 h after inoculation (HAI) with *S. sclerotiorum*; Figure S5: hypothetical representation of immune responses and signaling in EXLB:TIR19-OE lines.; Table S1: *Arachis stenosperma* libraries (RNA-Seq and 454) used in the study and their NCBI accession numbers; Table S2: primers used for PCR and qRT-PCR in this study; Table S3: truncated NLR (TNx) in *A. stenosperma* chromosome location, size and their orthologs in *A.duranensis*; Table S4: aminoacid substitutions between *A. stenosperma* (*AsTIR19*) and *A. duranensis* (Aradu.G29LA) predicted proteins. Refs [106,107,108,109,110,111,112,113,114,115,116] in Supplementary Materials.

## Abbreviations

ACC	aminocyclopropane-1-carboxylate deaminase
ACS	1-aminocyclopropane-1-carboxylate synthase
APX1	Ascorbate peroxidase
CA	Beta-Carbonic anhydrase
CAT	catalase
CC domain	Coiled-Coil Domain
CPKs	Calcium-dependent Protein Knases
CDS	Coding Sequence
DAI	Days after Inoculation
DEG	Differentially Expressed Gene
EDS1	Enhanced Disease Susceptibility1
EF26	Ethylene-forming enzyme
ET	Ethylene
ETI	Effector Triggered Immunity
FC	Fold Change
FDR	False Discovery Rate
GO	Gene Ontology
HAI	Hours after Inoculation
HR	Hypersensitive Response
HIN1	Harpin-induced gene 1
HSR515	Hypersensitivity-related 515
HSR201	Hypersensitivity-related 201
ID	Integrated Domains
J2	Second-stage Juveniles
JA	Jasmonic Acid
LRR	Leucine Rich Repeat
MAMPS	Microbe-Associated Molecular Pattern
MAPK	Mitogen-Activated Protein Kinase
NACHT	NTPase domain found in apoptosis proteins as well as those involved in MHC transcription activation
NAD	nicotinamide adenine nucleotide
NBS	nucleotide-binding site
NLR	Nucleotide-binding and Leucine-rich repeat LRR domains
NPR1	Nonexpressor of Pathogenesis-Related Genes 1
OA	Oxalic Acid
OE	Overexpression Lines
PAMPs	Pathogen Associated Molecular Patterns
PB1 domain	Phox/Bem1p
PK	Protein Kinase
PR	Pathogenesis related
PRR	Pattern Recognition Receptors
PTI	Patterns Triggered Immunity
qRT-PCR	quantitative Reverse Transcription-Polymerase Chain Reaction
RbohD	NADPH/respiratory burst oxidase protein D
RKN	Root-Knot Nematode
RLKs	Receptor-Like Kinases
RLM3	Leptosphaeria maculans 3 gene
RLPs	Receptor-Like Proteins
RPW8	Resistance to Powdery Mildew 8
RQ	Relative Quantification
ROS	Reactive Oxygen Species
SA	Salicylic Acid
SAR	Systemic Acquired Resistance
SDD	Submitted to dry-down
SND	Submitted to combined nematode and drought stress
SDHY	Submitted to dehydration
SN3, SN6, SN9	Submitted to nematode infection at 3, 6 and 9 Days After Infection
SNPs	Single Nucleotide Polymorphism
SUV	Submitted to UV exposur
SsPINE1	*Sclerotinia sclerotiorum* PGIP-Inactivating Effector 1
TaRCR1	*Triticum aestivum* RCR1 gene
TIR	Toll and IL-1 receptor
TF	Transcription Factor
TNL	TIR-NBS-LRR
TNx	TIR-NBS
UV	Ultraviolet
WT	Wild Type
