# Supplementary material for: Engineering Resistance against Sclerotinia sclerotiorum Using a Truncated NLR (TNx) and a Defense-Priming Gene
Source: Plants (Basel). 2022 Dec 13;11(24):3483. doi: 10.3390/plants11243483 (PMC9786959; doi:10.3390/plants11243483)
Supplement: Supplementary file 1 [file plants-11-03483-s001.zip › plants-2026940-Supplementary Materials.pdf]

Supplementary Materials:

Supplementary Figure S1. *AsTIR19:* A) genomic sequence- 2,284bp (- strand); B) CDS sequence- 1,920 bp; C) protein sequence – 639 amino acids.

```
TTACCTTATTATTTTCCAAAACATCACAAGCATCACCAACATCCCACAAATGGCTATGAC
TTTGACAATTCTTGATAGTTCTCTGACAAAGGATTCTCTACCCCATTTCTTGTACAAGAC
ATGAGATAACCCAAAATGATCGAACATAGCTATTAGAGCCTCATCAAGAAGGGAACTCAT
GCCTCTAGCTGTATAGAAACTGCAACTATCAAATACATTAACTAGTTGTTCCTTTTGCTC
CCCCTTAAAAACTGATGCAATGTCTAGAACAATATGATTCCCCTGATAATCTGATCCATT
ACAACTCAATCTTAACACATTTTGAATTGCTGCATTAGGATTCTTCTCGAGTTTTTGCAG
AACATTATCCCATTCAGTTTCACTCTTGGAAAAAAGAAAAGAACCCAATTCTTTTAAAGC
TAATGGGACACCTTTGCAGTAGGTAACTGCTTTCCATGATAGCTCTTGGTATCCCCTTTT
TGGATAGTCTTGATTGAACGCATTCAAGCTAAACAATATAAGGGAATCTTGAAAATCTAA
CACCTCCATCTGATGAAATTTTTCAACTCCTTTTTTCAGCACACTCTTGTCCCTTGTTAT
CACAATGATTCTACTTCCTGCACCATAGCACTGAAGGTCCCCAGCTAGGTATTCTAATTG
TTTTGAGCTACTCACATCATCAAGAACTATGAAATTTCTTTGTCTGCTGAGCCTGCACTT
TAAAGTTGTATTTGCCATGGTATTTTCGCAATCTTTTCCTTGCAACAACTCCAAGTAAAG
TTGATTTCGCAACTCATCTAGTCCCCGCCCATACTTTTCTGATCTTTCCCGAACATTTCC
TAGGAAGCAGGAACCTTCATACTGATGAGAAAGTTCCTGATATAAAAGTCTTGCTAAAGT
TGTTTTGCCCAATCCTCCCATCCCACAAATCCCAATTATTCCCGTTTGTTCATCAGGAAC
TGAACTTAATAACATTTCTAACTTTTCATGATTTTTATCAATACCAACTAAACTCTTGAC
ATCCTTATGCAGGCAATGATTATTCAGCTCTCGCAAAATATCTTTGACAATCTTTTCAAC
AAGTTCAAAGTCGTCCCTACAGTATGAATAAATAATTAAAAAAAAAAAAAAGAAACATAGC
CAGGAGCAGATAAGATTTACTGCATTATATAACCAGAAATTAAATTGAATTAAAAAGTCA
AAACAATTATTTCGTATTACAATCGATCATCTAAACAGCTATTTCTCAATTAAAAATAAT
TCATCCTTACCTACAAGAGCCGGAGTCCCAGCCATCTAAATCCGCTATTTTGGCGAGAGC
TTCCATCCATTTCAGAAGATTGTTAATGTCACACTGCAGGTGAGACTTGAATTTTTCATT
GAAACTTACTAGCTGCTTTCGCACATCTGATGGATCCACACCATAAAACACAGGTATAAC
TTTCCTTCCGCACTCCATTATTTTCAATAACTCGTGAAGGCACCAACTTGAGGTAACAAA
ATTCTCTGACAAGATGATAACAGATATAGACGAATTTTCAATAGCTCTTGTAAGAGAATC
CAAGATGCAATCCCCTTTGTGAAGCTCATCATCAATGAAGGTTTTTATTTGGTTTTGATT
CAGAGCTCCGACAAGATGGGAACTGAATCCCGTACGAATGTCCTTCCCTCTAAAGCTGAT
AAACACATCATACTTCCAAAGATTCGAAGATAGCCTATTAGAGTTTGGCTTAACCGAATC
GAGAGGTTTGTTAGGGTTTGGGAAGAGGAAGAGTCTCATGCGGGCGAATCCAGGGGAATC
GCGGTAGAGTTGATCGAACTCGAGCATCATGTTATGAAGGTCTCTGTCGTTGGTGACGGA
GACGAGAGCATTGAGGTCATCGCTGCCGGGGACTTGGTATTTGAAGAAGTTGTCGCGGTT
GGAGGCGCCGTCAAAAAGGCTGGAGATTTCGGCAACCATGGCTGTGAAGTCAATTCTGCG
GTCAACACGAAGAAGCTTATTGGTTCCATCGATGTAGGCGAGCTTGTTGTTGTGGCCGCG
GGGCTTGATCTCGCCGCCGTAGCTGCAGAGAAACTTTGCTTCGCGGATCGCCATGTAACT
GAACTTAGTATTTTGTATTCTGTGGTTTTGAGTTTGAAATTCGCATAGGCAACAGCAACT
TCAGTCGTTGAAATACTCCTTCTCTGACTGCGTGACCGGAAATTGCAAGATGACTTCCAC
GAAACCTCCAGGAGCGTTCGGCAACATCGGGTTTTGGGGTTTTTAAGTGAATGAAATTGT
TGAG
Grey- 5`UTR
```

(A)

ATGGCGATCCGCGAAGCAAAGTTTCTCTGCAGCTACGGCGGCGAGATCAAGCCCCGCGGCCACAACAACAAGCTCGCCT
ACATCGATGGAACCAATAAGCTTCTTCGTGTTGACCGCAGAATTGACTTCACAGCCATGGTTGCCGAAATCTCCAGCCT
TTTTGACGGCGCCTCCAACCGCGACAACTTCTTCAAATACCAAGTCCCCGGCAGCGATGACCTCAATGCTCTCGTCTCC
GTCACCAACGACAGAGACCTTCATAACATGATGCTCGAGTTCGATCAACTCTACCGCGATTCCCCTGGATTCGCCCGCA
TGAGACTCTTCCTCTTCCCAAACCCTAACAAACCTCTCGATTCGGTTAAGCCAAACTCTAATAGGCTATCTTCGAATCT
TTGGAAGTATGATGTGTTTATCAGCTTTAGAGGGAAGGACATTCGTACGGGATTCAGTTCCCATCTTGTCGGAGCTCTG
AATCAAAACCAAATAAAAACCTTCATTGATGATGAGCTTCACAAAGGGGATTGCATCTTGGATTCTCTTACAAGAGCTA
TTGAAAATTCGTCTATATCTGTTATCATCTTGTCAGAGAATTTTGTTACCTCAAGTTGGTGCCTTCACGAGTTATTGAA
AATAATGGAGTGCGGAAGGAAAGTTATACCTGTGTTTTATGGTGTGGATCCATCAGATGTGCGAAAGCAGCTAGTAAGT
TTCAATGAAAAATTCAAGTCTCACCTGCAGTGTGACATTAACAATCTTCTGAAATGGATGGAAGCTCTCGCCAAAATAG
CGGATTTAGATGGCTGGGACTCCGGCTCTTGTAGGGACGACTTTGAACTTGTTGAAAAGATTGTCAAAGATATTTTGCG
AGAGCTGAATAATCATTGCCTGCATAAGGATGTCAAGAGTTTAGTTGGTATTGATAAAAATCATGAAAAGTTAGAAATG
TTATTAAGTTCAGTTCCTGATGAACAAACGGGAATAATTGGGATTTGTGGGATGGGAGGATTGGGCAAAACAACTTTAG
CAAGACTTTTATATCAGGAACTTTCTCATCAGTATGAAGGTTCCTGCTTCCTAGGAAATGTTCGGGAAAGATCAGAAAA
GTATGGGCGGGGACTAGATGAGTTGCGAAATCAACTTTACTTGGAGTTGTTGCAAGGAAAAGATTGCGAAAATACCATG
GCAAATACAACTTTAAAGTGCAGGCTCAGCAGACAAAGAAATTTCATAGTTCTTGATGATGTGAGTAGCTCAAAACAAT
TAGAATACCTAGCTGGGGACCTTCAGTGCTATGGTGCAGGAAGTAGAATCATTGTGATAACAAGGGACAAGAGTGTGCT
GAAAAAAGGAGTTGAAAAATTTCATCAGATGGAGGTGTTAGATTTTCAAGATTCCCTTATATTGTTTAGCTTGAATGCG
TTCAATCAAGACTATCCAAAAAGGGGATACCAAGAGCTATCATGGAAAGCAGTTACCTACTGCAAAGGTGTCCCATTAG
CTTTAAAAGAATTGGGTTCTTTTCTTTTTTCCAAGAGTGAAACTGAATGGGATAATGTTCTGCAAAAACTCGAGAAGAA
TCCTAATGCAGCAATTCAAAATGTGTTAAGATTGAGTTGTAATGGATCAGATTATCAGGGGAATCATATTGTTCTAGAC
ATTGCATCAGTTTTTAAGGGGGAGCAAAAGGAACAACTAGTTAATGTATTTGATAGTTGCAGTTTCTATACAGCTAGAG
GCATGAGTTCCCTTCTTGATGAGGCTCTAATAGCTATGTTCGATCATTTTGGGTTATCTCATGTCTTGTACAAGAAATG
GGGTAGAGAATCCTTTGTCAGAGAACTATCAAGAATTGTCAAAGTCATAGCCATTTGTGGGATGTTGGTGATGCTTGTG
ATGTTTTGGAAAATAATAAGGTAA

(**B**)

MAIREAKFLCSYGGEIKPRGHNNKLAYIDGTNKLLRVDRRIDFTAMVAEISSLFDGASNRDNFFKYQVPGSDDLNALVS
VTNDRDLHNMMLEFDQLYRDSPGFARMRLFLFPNPNKPLDSVKPNSNRLSSNLWKYDVFISFRGKDIRTGFSSHLVGAL
NQNQIKTFIDDELHKGDCILDSLTRAIENSSISVIILSENFVTSSWCLHELLKIMECGRKVIPVFYGVDPSDVRKQLVS
FNEKFKSHLQCDINNLLKWMEALAKIADLDGWDSGSCRDDFELVEKIVKDILRELNNHCLHKDVKSLVGIDKNHEKLEM
LLSSVPDEQTGIIGICGMGGLGKTTLARLLYQELSHQYEGSCFLGNVRERSEKYGRGLDELRNQLYLELLQGKDCENTM
ANTTLKCRLSRQRNFIVLDDVSSSKQLEYLAGDLQCYGAGSRIIVITRDKSVLKKGVEKFHQMEVLDFQDSLILFSLNA
FNQDYPKRGYQELSWKAVTYCKGVPLALKELGSFLFSKSETEWDNVLQKLEKNPNAAIQNVLRLSCNGSDYQGNHIVLD
IASVFKGEQKEQLVNVFDSCSFYTARGMSSLLDEALIAMFDHFGLSHVLYKKWGRESFVRELSRIVKVIAICGMLVMLV
MFWKIIR-

(**C**)

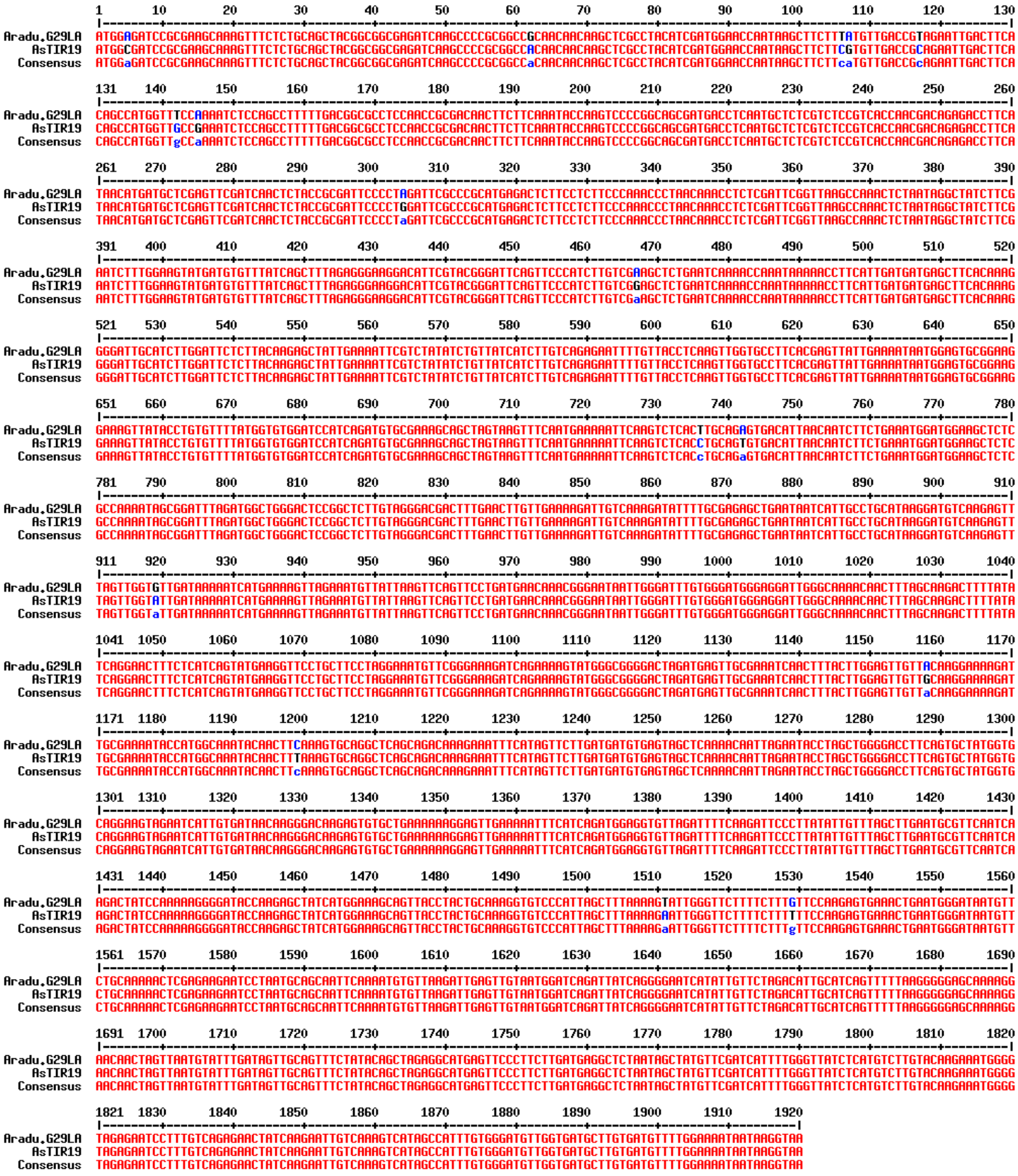

(a)

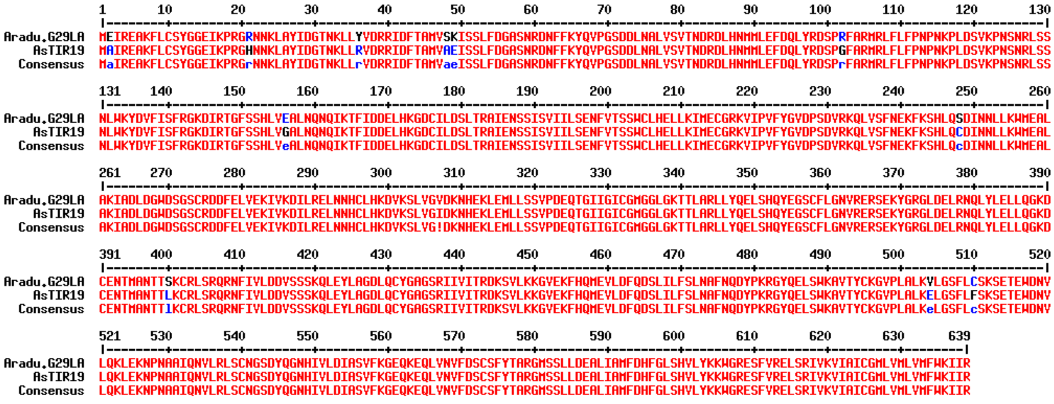

(b)

Supplementary Figure S2. Sequence alignment of *A. duranensis* (*Aradu. G29LA*) and *A. stenosperma* (*AsTIR19*): a) coding sequences (CDS) showing 16 SNPs; b) predicted proteins showing 11 aminoacids substitutions

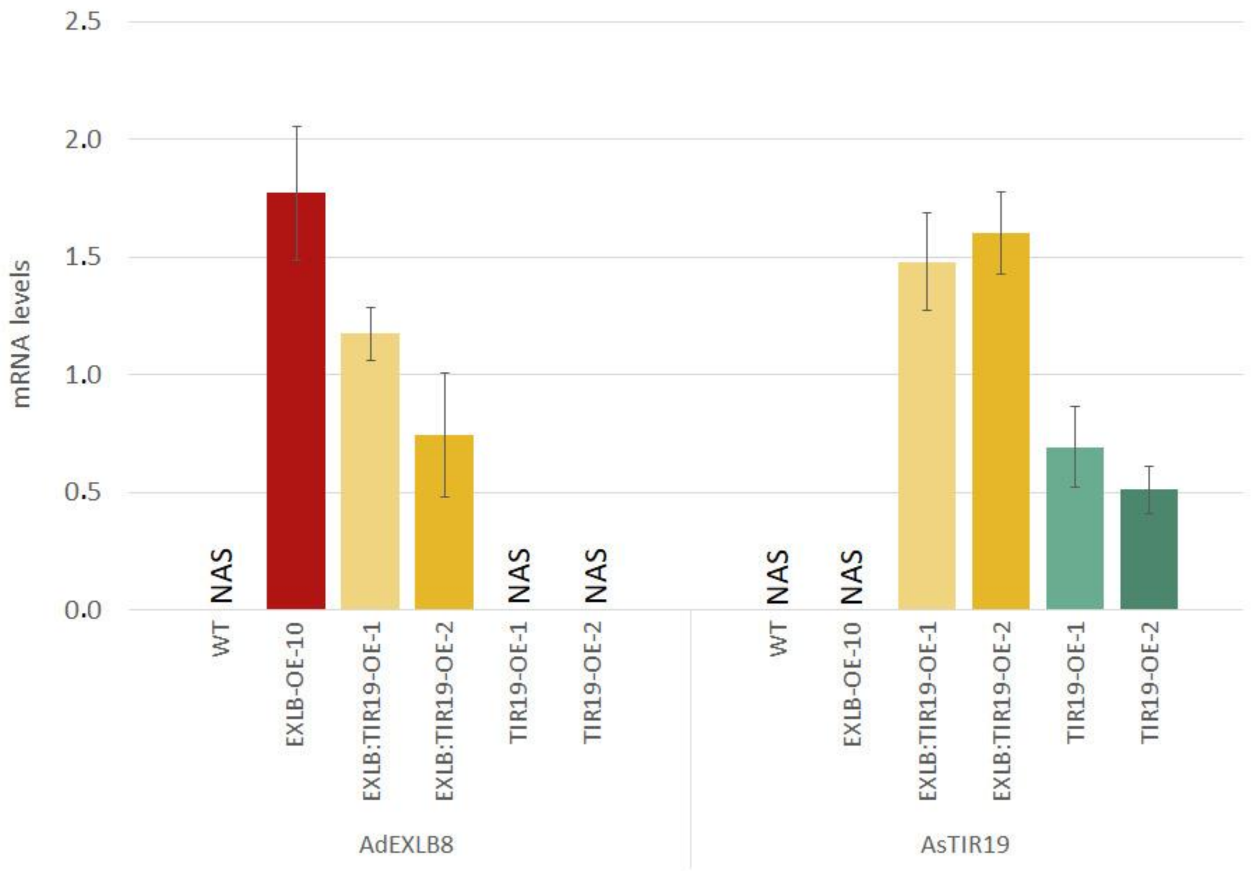

Supplementary Figure S3. qRT-PCR analysis of basal expression of *AsTIR19* and *AdEXLB* transgenes in the five independent tobacco OE lines and wild-type.

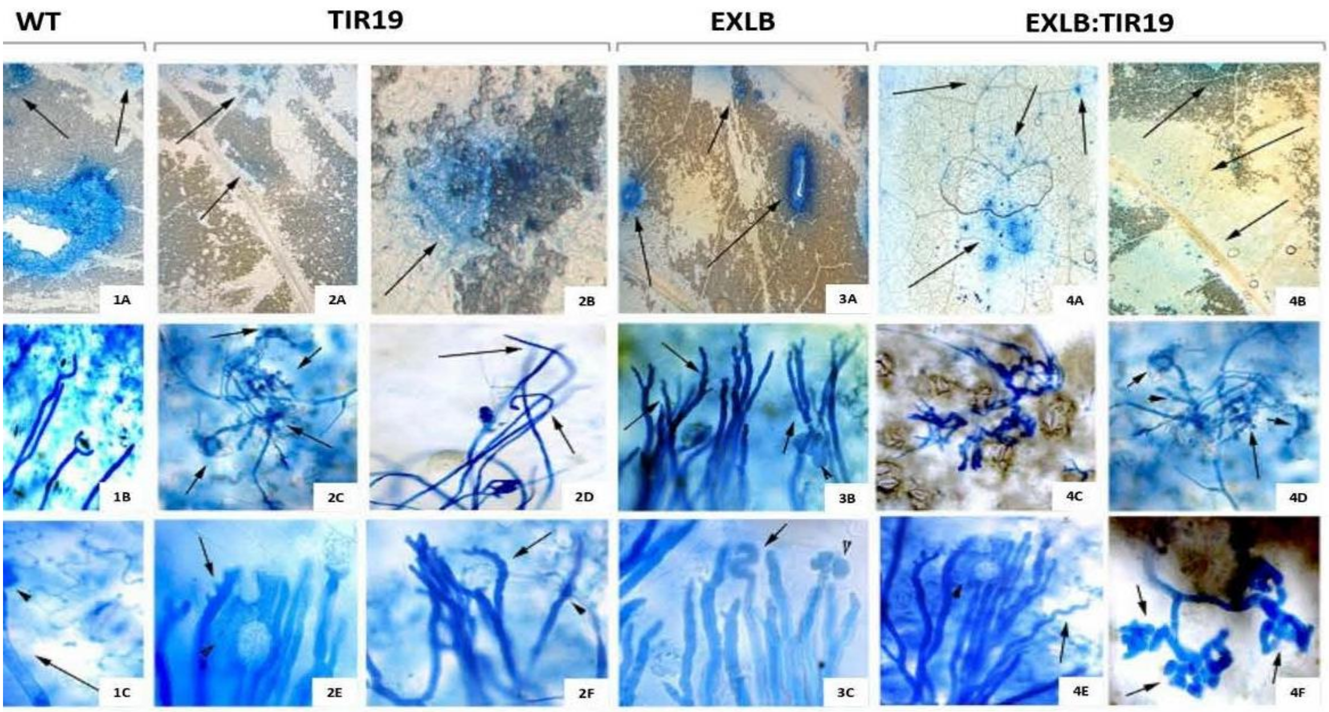

Supplementary Figure S4. Detached leaves of *tobacco* OE lines and WT stained by cotton blue and collected at 10 and 14 hours after inoculation (HAI) with *S. sclerotiorum*. **1)** WT leaves showing regular hyphal growth: **(1a)** hyphae growth surrounding the agar plug region; **(1b)** elongated hyphae growth; **(1c)** elongated and enlarged hyphae forming cushion (arrowhead); **2)** *TIR19* OE lines (#1 and #2): **(2a)** irregular hyphae growth; **(2b)** small and faded blue area due to the low mycelia growth; **(2c,2d)** morphological altered hyphae; mycelia showing misdirected growth; **(2e,2f)** hyphae with branches and cushions showing an irregular morphology (arrowhead); **3)** *EXLB8* OE line: **(3a)** small region stained in blue (arrows); **(3b, 3c)** hyphae with contorted and branched hyphae (arrows) and irregular formation of cushion (arrow)**; 4)** *EXLB8: TIR19* OE lines (#1 and #2) : **(4a,4b)** no detectable or small regions stained in blue (arrows); **(4c,4d,4e,4f)** contorted and branched disorganized hyphae (arrows) irregular formation of cushion (arrowhead). Detached leaves of tobacco OE lines and WT stained by the cotton blue and collected at 10 and 14 hours after inoculation (HAI) with *S. sclerotiorum*.

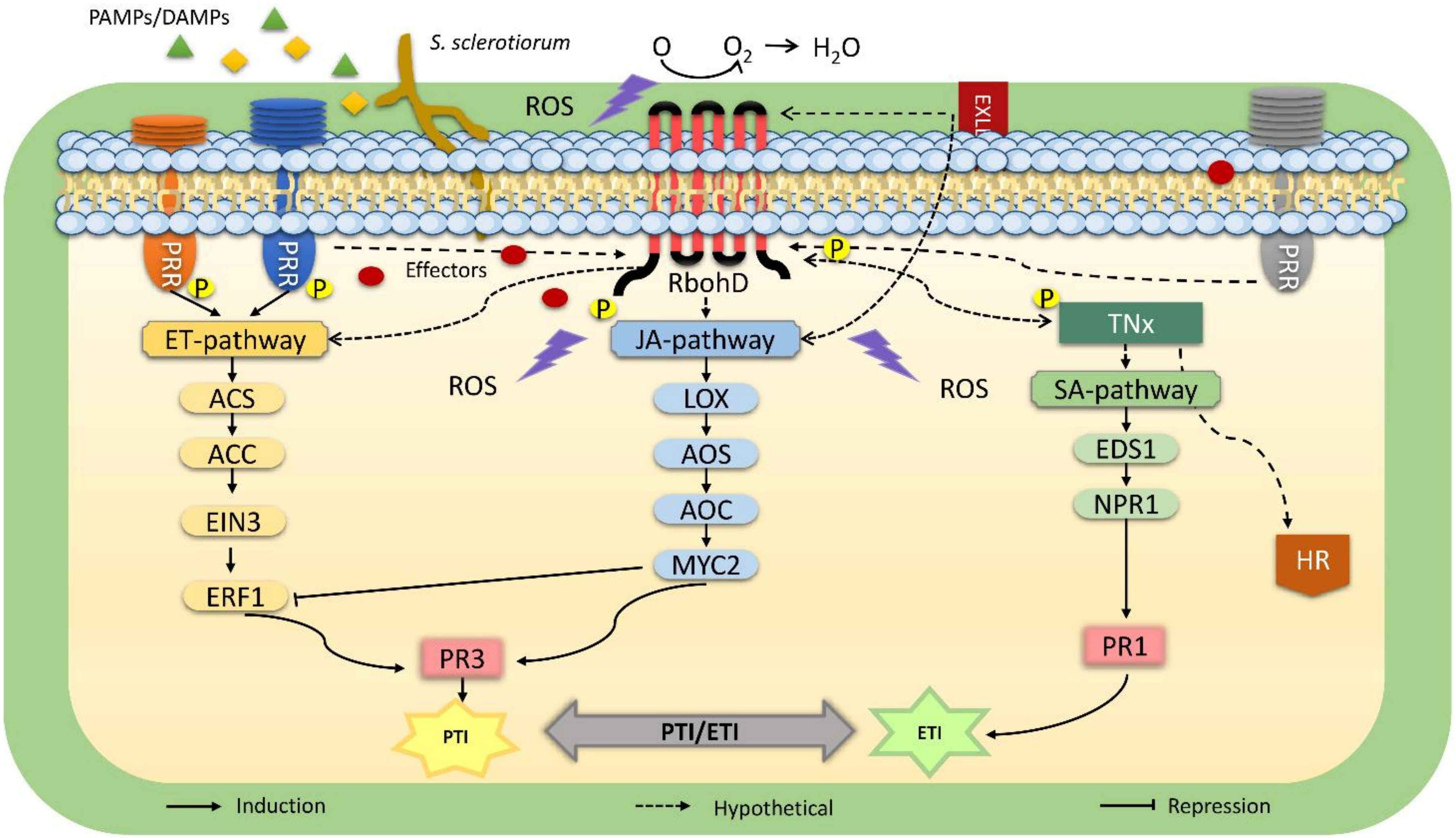

Supplementary Figure S5. Hypothetical representation of immune responses and signaling in EXLB:TIR19 -OE lines.

Supplementary Table S1. *Arachis stenosperma* libraries (RNA-Seq and 454) used in the study and their NCBI accession numbers.

Stress	Library	Total reads	Reference	NCBI accession
RNASeq-Illumina				
Meloidogyne arenaria	SNC- control	6,651,071	[47]	SAMN03734821
Meloidogyne arenaria	SN3-3DAI	7,162,500	[47]	SAMN03734822
Meloidogyne arenaria	SN6 -6DAI	6,333,182	[47]	SAMN03734823
Meloidogyne arenaria	SN9- 9DAI	6,202,969	[47]	SAMN03734824
Drought	SDD- control	100,326,990	[101]	SAMN16049051
Drought	SDD-7DAI	112,787,062	[101]	SAMN16049053
UV-C exposure	SUVC -control	36,001,334	[50]	SAMN22043423
UV-C exposure	SUV- (25-150min)	38,886,104	[50]	SAMN22043424
Dehydration	SDHY (0 min)	12,219,081	[51]	SAMN08225801
Dehydration	SDHY (25-150)	12,587,737	[51]	SAMN08225802
Cross-stress	SND	107,186654	[101]	SAMN16049054
454 sequencing				
Cercosporidium personatum	SFUC- control	168,555	[46]	SAMN00740097
Cercosporidium personatum	SFU- 7DAI	194,076	[46]	SAMN00740097

Supplementary Table S2. Primers used for PCR and qRT-PCR in this study.

Primer name	Putative function	Forward primer (5'-3')	Reverse primer (5'-3')	Amplicon (bp)	Efficiency of primer	Reference
Arachis spp.						
60S	60S ribosomal protein L10	TCTTTTGACGACCAGGGAAC	TGGAGTGAGAGGTGCATTTG	155	0,86	[100]
GAPDH	Glyceraldehyde-3-phosphate dehydrogenase	ATCACTGCCACCCAGAAAAC	CAACAACGGAGACATCAACG	190	0,84	[100]
AdEXLB8	Expansin-like B	TGGGTTCTCAACATCAAACACT	CGCAGCTACAACCCACACTA	150	0,84	[106]
AsTIR19	Disease resistance protein	GGCATGAGTTCCCTTCTTGA	CACAAGCATCACCAACATCC	159	0,86	This study
**Bacterial transformation genotyping**						
eGFP	Green Fluorescent Protein	CGACCACATGAAGCAGCACGAC	TCCTCGATGTTGTGGCGGATCT	294	-	[107]
BAR	Glufosinate ammonium	AAACCCACGTCATGCCAGTT	CATCGAGACAAGCACGGTCA	405	-	[108]
Nicotiana tabacum						
**Reference genes**						
NtL25	Ribosomal protein L25	GCTAAGGTTGCCAAGGCTGTCAAG	GCACTAATACGAGGGTACTTGGGGTTT	133	0,88	[52]
NtActin	Actin	CATTGGCGCTGAGAGATTCC	GCAGCTTCCATTCCGATCA	68	0,88	[109]
**Salicylic Acid (SA)**						
NtEDS1	Enhanced Disease Susceptibility 1	TGATGACTTCAATGCTAATGTGAG	GATCATGTAAGGTCCTGTATCTTC	214	0,86	[110]
NtNPR1	Nonexpressor of Pathogenesis-relared genes 1	GAATGATACGGCAGAAGA	AGATGAGGAGATGTTGTTAG	124	0,84	[111]
NtPR1	Pathogenesis related protein-1	AACCTTTGACCTGGGACGAC	GCACATCCAACACGAACCGA	271	0,86	[110]
NtPR4	Pathogenesis related protein-4	GGAAAACGGAAAGGTAAGAAGAGG	GGACACGAGGTAGGTATCACAACAA	222	0,85	[110]
**Hypersensitivity Reaction (HR)**						
NtHIN1	Harpin-induced gene 1	CGACCTAACAAAGTCAAGTTCTACG	CTCTATCTCCCAATAAAACCAAGC	283	0,84	[112]
NtHSR201	Hypersensitivity-related 201	CAGCAGTCCTTTGGCGTTGTC	GCTCAGTTTAGCCGCAGTTGTG	173	0,85	[112]
NtHSR515	Hypersensitivity-related 515	TTGGGCAGAATAGATGGGTA	TTTGGTGAAAGTCTTGGCTC	499	0,83	[110]
**Jasmonic Acid (JA)**						
NtLOX	Linoleate 9S-lipoxygenase 5	GGACTTGAAGGATGTTGGTGC	TCACATTAAACGTAGCATCTCCT	199	0,89	[113]
NtAOS	Allene oxide synthase	GCCAAACGCGACCTTATGAT	CCACAAAATCCTTTCCGGCA	249	0,85	[113]
NtAOC	Allene oxide cyclase	CCTGCTTATCTTCGCTTGAG	ATGCAGAGTCCAGCCGTTAT	121	0,86	[114]
NtMYC2	Transcription factor MYC2	CCTCATTGATGGTGCTCGTG	TTTCGGTGTTCTTGCTCAGC	182	0,88	[113]
**Reactive Oxygen Species (ROS)**						
NtCA	Beta-Carbonic anhydrase	CGCCTGTGGAGGTATCAAA	GAGAAGGAGAAAGACCGAACT	116	0,83	[109]
NtCAT1	Catalase 1	TGGACTTCATACTGGTCTCA	TTCCCATTGTTTCAGTCATTCA	275	0,85	[109]
NtAPX1	Ascorbate peroxidase	GAGAAATATGCTGCGGATGA	CGTCTAATAACAGCTGCCAA	235	0,83	[109]
NtRbohD	Respiratory burst oxidase homologue D	ACCAGCACTGACCAAAGAA	TAGCATCACAACCACAACTA	237	0,85	[109]
**Ethylene (ET)**						
NtACC	1-aminocyclopropane-1-carboxylate deaminase	TCTGAGGTTACTGATTTGGATTGG	TGGACATGGTGGATAGTTGCT	264	0,91	[115]
NtACS6	1-aminocyclopropane-1-carboxylate synthase	ATGCCAAGGAAAGGGATTCTACA	TGGGAGGTTTGGGCGAAGA	132	0,90	[115]
NtACO	1-aminocyclopropane-1-carboxylate oxidase	GACAAAGGGACATTACAAGAAGT	GAGAAGGATTATGCCACCAG	403?	0,84	[115]
NtEFE26	Ethylene-forming enzyme	CGGACGCTGGTGGCATAAT	CAACAAGAGCTGGTGCTGGATA	267	0,86	[115]
NtEIN3	Ethylene insensitive 3	AAATGGACCTGCAGCCATAG	TGAAGCTCCTGCAAAGTGTG	108	0,93	[116]
NtERF1	Ethylene-responsive transcription factor 1	TTAACGTCGGATGGTCGCCG	ACACCTCTGTAATGCCTTCC	142	0,9	[114]
NtPR3	Pathogenesis related protein-3	CAGGAGGGTATTGCTTTGTTAGG	CGTGGGAAGATGGCTTGTTGTC	222	0,85	[110]

Supplementary Table S3. Truncated NLR (TNx) in *A. stenosperma* chromosome location, size and their orthologs in *A.duranensis*.

A. stenosperma	Chromossome	Scafold	Size	*A.duranensis*
AsTir19	chr09	CM026098.1:8528033-8530316	2,284bp	Aradu.G29LA
AsTir24	chr04	CM026093.1:149025659-149028454	2,796bp	Aradu.F12KD
AsTir31	chr05	CM026094.1:115464892-115466608	1,717bp	Aradu.24NBV
AsTir37	chr05	CM026094.1:115578999-115581705	2,707bp	Aradu.NL1YQ
AsTir41	chr08	CM026097.1:46704525-46705492	968bp	Aradu.HLR71
AsTir53	chr04	CM026093.1:148165998-148168440	2,443bp	Aradu.4QC4Y
AsTir54	chr02	CM026091.1:99318774-99322300	3,527bp	Aradu.87VP9
AsTir55	chr02	CM026091.1:14377144-14380571	3,428bp	Aradu.9435V
AsTir56	chr08	CM026097.1:24761678-24766836	5,159bp	AsTir54
AsTir57	chr09	CM026098.1:8434052-8435449	1,398bp	Aradu.B1RD2
AsTir58	chr04	CM026093.1:147950757-147953010	2,254bp	Aradu.B45Z7
AsTir59	chr09	CM026098.1:8424536-8425745	1,210bp	Aradu.C9QSM
AsTir60	chr04	CM026093.1:148849084-148851557	2,474bp	Aradu.E1LE1
AsTir61	chr02	CM026091.1:99150710-99153366	2,657bp	Aradu.E2CQ9
AsTir62	chr04	CM026093.1:148891917-148895237	3,321bp	Aradu E48FH
AsTir63	chr08	CM026097.1:24942115-24943660	1,546bp	Aradu.G2TQN
AsTir64	chr02	CM026091.1:99396811-99399908	3,098bp	Aradu.HQX6B
AsTir65	chr05	CM026094.1:986178-991924	5,747bp	Aradu.JFT50
AsTir66	chr04	CM026093.1:149001690-149004512	2,823bp	Aradu.MC6ZY
AsTir67	chr08	CM026097.1:36632785-36634889	2,105bp	Aradu.MT30J
AsTir68	chr09	CM026098.1:8424536-8425745	1,210bp	Aradu.RT9LH
AsTir69	chr04	CM026093.1:148572691-148575490	2,800bp	Aradu.S2WWI
AsTir70	chr04	CM026093.1:148165998-148168752	2,755bp	Aradu.ULV47
AsTir71	chr09	CM026098.1:8405163-8407619	2,457bp	Aradu.ZG5UW

Supplementary Table S4. Aminoacids substitutions between *A. stenosperma* (*AsTIR19*) and *A. duranensis* (*Aradu.G29LA*) predicted proteins.

Amino acid position	*A. stenosperma*	*A. duranensis*	Substitution
2	Alanine - A	Glutamate - E	Conservative
21	Histidine - H	Arginine - R	Conservative
36	Arginine - R	Tyrosine - Y	Conservative
48	Alanine - A	Serine - S	Non-conservative
49	Glutamic acid - E	Lysine - K	Non-conservative
102	Glycine - G	Arginine - R	Conservative
156	Glycine - G	Glutamic acid - E	Conservative
248	Cysteine - C	Serine - S	Conservative
307	Isoleucine - I	Valine - V	Non-conservative
400	Leucine - L	Serine - S	Conservative
504	Glutamate - E	Valine - V	Conservative
510	Phenylalanine - F	Cysteine - C	Conservative
